# Cementing of the hip arthroplasty stem increases load-to-failure force: a cadaveric study

**DOI:** 10.1080/17453674.2019.1634331

**Published:** 2019-07-08

**Authors:** Antonio Klasan, Martin Bäumlein, Christopher Bliemel, Sven Edward Putnis, Thomas Neri, Markus Dietmar Schofer, Thomas Jan Heyse

**Affiliations:** a University Hospital Marburg, Center for Orthopedics and Traumatology, Marburg, Germany;; b Sydney Orthopaedic Research Institute, Chatswood, Australia;; c University Hospital St. Etienne, Department of Orthopaedic Surgery, Saint-Priest-en-Jatez, France;; d Orthomedic Frankfurt Offenbach, Offenbach, Germany

## Abstract

Background and purpose — To date, there is not a single clinical or mechanical study directly comparing a cemented and a cementless version of the same stem. We investigated the load-to-failure force of a cementless and a cemented version of a double tapered stem.

Material and methods — 10 femurs from 5 human cadaveric specimens, mean age 74 years (68–79) were extracted. Bone mineral density (BMD) was measured using peripheral quantitative computed tomography. None of the specimens had a compromised quality (average T value 0.0, –1.0 to 1.4). Each specimen from a pair randomly received a cemented or a cementless version of the same stem. A material testing machine was used for lateral load-to-failure test of up to a maximal load of 5.0 kN.

Results — Average load-to-failure of the cemented stem was 2.8 kN (2.3–3.2) and 2.2 kN (1.8–2.8) for the cementless stem (p = 0.002). The cemented version of the stem sustained a higher load than its cementless counterpart in all cases. Failure force was not statistically significantly correlated to BMD (p = 0.07).

Interpretation — Implanting a cemented version of the stem increases the load-to-failure force by 25%.

It has been shown that cementless hip arthroplasty components have a higher incidence of periprosthetic femoral fractures (PFFs), with type 2 stems the most susceptible (Carli et al. 2017). The type 2 stem is calcar loading and the most commonly used (Khanuja et al. 2011). Cemented stems are primarily classified as loaded-taper or composite-beam (Scheerlinck and Casteleyn 2006) with the former having a reported higher PFF incidence rate (Carli et al. 2017).

Mechanically, only loaded-taper designs have been investigated for load-to-failure. Ginsel et al. (2015) found that an Exeter stem (Stryker Orthopaedics, Mahwah, NJ, USA) with a larger cross-section tolerates more torque until failure, Morishima et al. (2014) found that an Exeter stem (Stryker) with increased length tolerates more torque and energy until failure. Erhardt et al. (2013) compared the double taper CPT stem (Zimmer, Warsaw, IN, USA) with the triple taper C-Stem (DePuy, Raynham, MA, USA) and found no difference in torque or energy, but only in fracture patterns. Clinically, both in electively implanted setting and in fracture treatment settings, cemented stems have a substantially lower incidence of PFF (Carli et al. 2017).

Due to different classifications of cementless and cemented stem designs, different availability of each stem in different countries as well as the fact that not all stem designs have a cemented and a cementless version, we were unable to identify a mechanical comparison of a cemented and cementless version of the same stem (Carli et al. 2017).

We investigated and quantified the direct load-to-failure force of a cementless and a cemented version of a double tapered stem. Based on current literature evidence, our hypothesis was that the cementless version would have lower load-to-failure.

## Material and methods

### Specimen preparation

The femurs were donated by the authors’ anatomical institute. The specimens originated from 4 male and 1 female adults with an average age of 74 years (68–79). After obtaining the paired femurs they were embalmed with a solution consisting of 96% ethanol and 2% formaldehyde. During perfusion, approximately 15 L of the solution was passed through the femoral artery. All specimens were thawed at thermostat temperature of 19 °C and the implantation and testing were performed at that same ambient temperature.

To exclude damage related to preexisting fractures or tumors, all specimens were clinically and radiographically examined for integrity. The surrounding soft tissue was stripped from the specimens. Bones were then wrapped in moist towels using the aforementioned embalming solution and stored in a cooling chamber at 4 °C to avoid drying artifacts until testing, which occurred after 6–12 months.

### Bone mineral density assessment

In order to exclude osteoporotic specimens and to do a comparison of load-to-failure and bone density, a bone mineral density (BMD) analysis was performed. Peripheral quantitative computed tomography (pQCT) measurements were used to analyze BMD. For the pQCT measurements, a Stratec XCT Research SA instrument was used (Stratec Medizintechnik GmbH, Pforzheim, Germany). Measurements of BMD were performed at the neck region after obtaining specimens from the cadavers.

### Femoral stem tested

**Figure 1.**
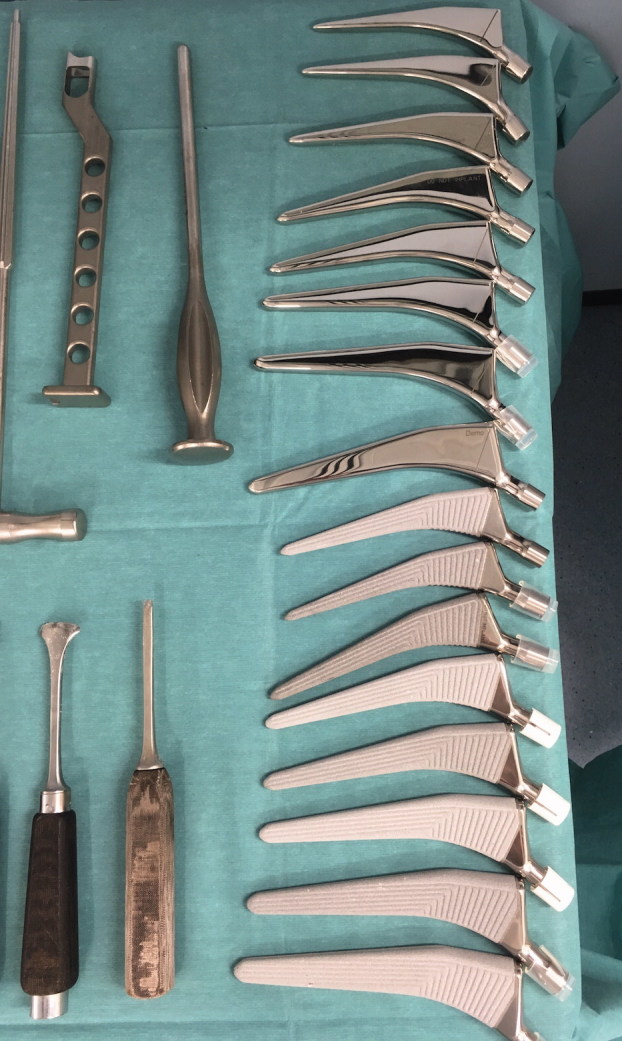
Cemented and cementless Polarstem with the original instruments.

The implants we compared were the cementless Polarstem and the cemented Polarstem (Smith & Nephew, Baar, Switzerland). Cementless Polarstem is a titanium alloy (Ti-6Al-4V ISO 5832-3) double tapered femoral stem with 180 µm of Ti-plasma spray combined with 50 µm of hydroxyapatite coating with fixation occurring on the calcar and metaphysis. Cemented Polarstem is a stainless-steel ISO 5832-9 double tapered femoral stem. We used standard stems with CCD 135°, with each specimen in a pair randomly receiving either a cemented or a cementless stem (Figure 1).

**Figure 2.**
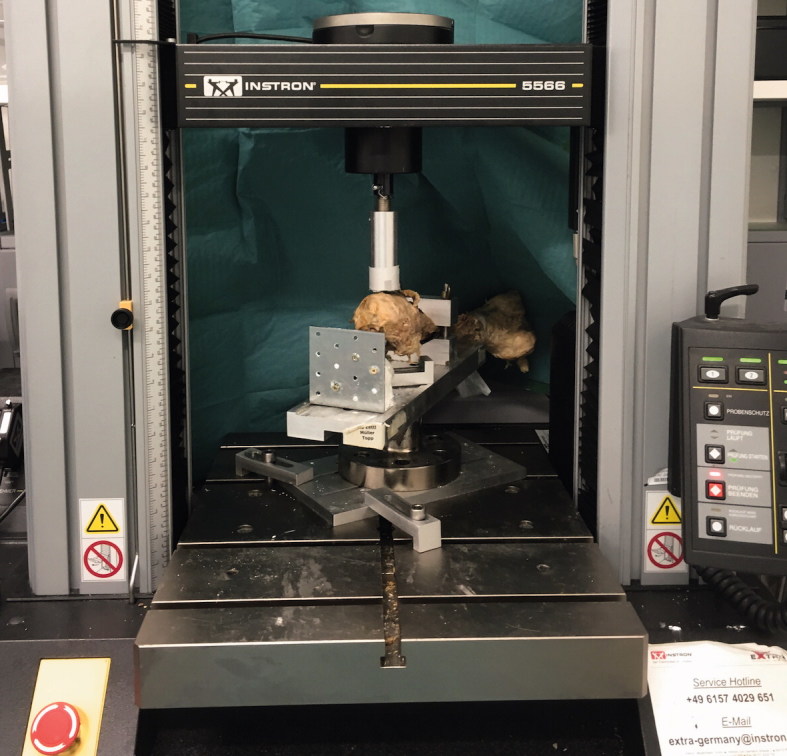
The mechanical setup.

The implantation was performed according to the manufacturer’s operating manual and using the original instruments. The trials were implanted until a secure press-fit was obtained. The trials were controlled radiologically for size and fracture. For the cemented version, we used 40 mL of Palacos R + G (Heraeus, Hanau, Germany) in a third-generation technique, with a Buck cement restrictor (Smith & Nephew, Baar, Switzerland). A minimum of 20 minutes was allowed for the cement to set. A polyethylene cup with an inner diameter of 32 mm was used as the acetabulum (Reflection Smith & Nephew, Baar, Switzerland). It was fixed with cement and screws in 45° inclination and 10° of anteversion. A 32 mm ceramic head (Biolox, Ceramtec, Plochingen, Germany) was implanted on the femoral component. The distal femoral fixation was placed at 40 cm distal from the resection using a screw clamp to prevent axial rotation of the specimen. Proximally, a joint was created by inserting the ceramic head into the created acetabulum. The femoral mechanical axis was set parallel to the ground (Figure 2).

### Load to failure assessment

Each specimen was tested with load-to-failure on an Instron 5566 universal testing machine (Instron Corp., Darmstadt, Germany) (Figure 2). A 3 cm diameter cylinder was attached to the testing machine and used to apply compression force axially. The test sequence started at 5 N force with the cylinder positioned directly over the greater trochanter. The load was continuously raised at a velocity of 3 N/s. Criteria for discontinuation of testing were premature rotation of the femur (fixation failure) or occurrence of a fracture (final result).

### Power analysis and statistics

First, a trial run with 4th-generation composite femurs (Sawbones, Pacific Research Laboratories, Inc., Vashon, WA, USA) was performed in the same manner as described above for the purpose of power analysis. Based on the differences in forces observed, to reach an alpha of 0.05 and a power of 0.8 the number of cadaveric pairs necessary was 5.

The data were collected at 100 ms intervals using the instrument-specific Bluehill Software (Instron, Norwood, MA, USA).

Load at failure (kN), and time (s) were recorded. The difference in force between implants was statistically analyzed using a paired t-test and the correlation of force and BMD was analyzed using Pearson’s correlation. Statistical significance was set at p < 0.05.

### Ethics, funding and potential conflicts of interest

All donors provided written consent by their own free will for the use of their body for research purposes. The study was approved by our institution’s ethics board (164/17, 02 November 2017). This study received no external funding.

AK has received research support from Implantcast. MS has been paid for presentations by DePuy/Synthes and Smith & Nephew. TH has been paid for presentations for Smith & Nephew, Zimmer Biomet, and Implantcast. He has received research support from Smith & Nephew, Zimmer Biomet, and Implantcast. He is a consultant to Smith & Nephew. Other authors have no conflicts of interest to declare.

## Results

### Bone mineral density

Bone mineral density of the tested femurs was 0.9 g/cm^2^ (0.8–1.1). There was no statistically significant difference in BMD between the specimens receiving cemented and cementless components (p = 0.8). None of the specimens were of compromised bone quality compared with the corresponding reference value (T-value range –1.0 to 1.4).

### Implants and load-to-failure

**Figure 3.**
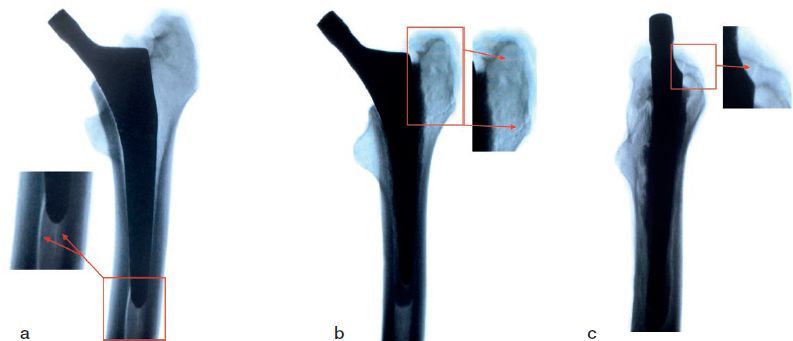
(a) Radiograph of the fracture of the femur with a cementless stem, anteroposterior view. (b) Radiograph of the fracture of the femur with a cemented stem, anteroposterior view. (c) Radiograph of the fracture of the femur with a cemented stem, lateral view.

Implant sizes were the same in each pair with 1 pair size 1, 2 size 3, 1 size 4, and 1 size 5. A fracture was produced consistently in all specimens. Average load-to-failure of the cemented stem was 2.8 kN (2.3–3.2). Average load-to-failure of the cementless stem was 2.2 kN (1.8–2.8). Cementless stems suffered a fracture of the medial wall on the level of the fracture, extending distally. Cemented stems fractured primarily on the greater trochanter, extending onto the lateral wall, which ultimately caused a dislocation of the stem (Figure 3). The cemented stems sustained a higher load than the cementless stems in all pairs. The mean estimated difference in force between the 2 stems was 0.6 kN (95% CI 0.3–0.8), which corresponds to a force produced by 57 kg. This difference was statistically significant (p = 0.002). There was no statistically significant correlation between fracture force and BMD (p = 0.07). 

## Discussion

This study demonstrates on average 25% increased load-to-failure force of a cemented version compared with a cementless version of the same femoral stem.

There are a number of studies comparing PFF rates in cemented and cementless stems after both elective total arthroplasty and hemiarthroplasty due to femoral neck fracture, but of different designs. After elective arthroplasty, an increased PFF incidence in cementless compared with cemented stems, with a 3- to 14-fold difference in incidence, was found in a number of studies (Cook et al. 2008, Sheth et al. 2013, Thien et al. 2014, Abdel et al. 2016). The highest reported incidence is nevertheless only 0.45% after 2 years (Thien et al. 2014) and 3.5% after 20 years (Abdel et al. 2016) for uncemented stems. After hemiarthroplasty due to femoral neck fracture, the differences are even more extreme and – for some cementless stems – concerning. The differences occur mainly due to poorer bone quality observed in hemiarthroplasty patients (Langslet et al. 2014). In their respective studies, Langslet et al. (2014), Inngul et al. (2015), Phillips et al. (2013), Foster et al. (2005) all uniformly report a higher incidence of PFF in cementless stems, with incidence of PFF with a cementless stem as high as 12% after 12 months (Inngul et al. 2015). Even young patients have been reported to have excellent outcomes after cemented stems (Kiran et al. 2018).

Mechanically, it has been shown that larger stems can improve primary stability of both cemented (Ginsel et al. 2015) and cementless (Fottner et al. 2017) stems due to a better bone load. Increasing the length of the cemented stem has also been shown to increase primary stability (Morishima et al. 2014). Both of these aspects increase the surface contact area between the implant and the bone and have been utilized in designing the stem used in this study (Klasan et al. 2018), when compared with the implant it was based on, the Corail stem (DePuy, Raynham, MA, USA). These 2 stems have not been tested against each other. Furthermore, Carli et al. (2017) report a lack of comparison between a cemented and a cementless version of the same stem with regards to PFF rate or load-to-failure. Data suggest that bone mineral density around the stem decreases more with a cemented stem (Li et al. 2007) than with a cementless stem (Flatøy et al. 2016, Aro et al. 2018), without a correlation to subsidence. Again, a direct comparison like that in this study is yet to be performed.

In clinical settings, the evidence suggests a higher risk of PFF (Hailer et al. 2010, Sidler-Maier and Waddell 2015, Carli et al. 2017) and a higher revision rate (McMinn et al. 2012) for cementless stems. Yet, the evidence suggests cementless stems are used more often (Lehil and Bozic 2014). According to some researchers, a clear consensus on when to use cemented stems is missing (Moskal et al. 2016). This lack of consensus and data was one of the reasons to perform this study. Due to a higher risk of revision in older patients with cementless stems (Jämsen et al. 2014), studies set different thresholds for “older age” patients (Moskal et al. 2016), which affects the results depending on the study population. Since the definition of “elderly” is also unclear in the orthopedic literature (Sabharwal et al. 2015), these thresholds are even more difficult to define. There is also an expectation of older patients having lower bone quality (Wright et al. 2014) where a combination of a higher risk of falling, soft bone, and slower osseointegration make the surgeon incline toward cementing the stem. This provides immediate osseointegration and protects the implant at the same time, as was shown in our study. In this study, where non-osteoporotic bones were used, the difference in force-to-failure corresponds to 80% of the weight of an average European adult (Walpole et al. 2012).

Stem design has also been shown to influence the PFF and revision rate for both cemented (Palan et al. 2016, Kazi et al. 2019) and cementless stems (Carlson et al. 2017). Also, cemented and cementless versions of the same stem do not exist as often or do not have worldwide distribution. For instance, the cemented version of the stem used in our study is not available in the United States despite showing excellent clinical results (Klasan et al. 2018). Further studies with other stems available in both versions in a clinical and a mechanical setting are needed to provide this information.

Several limitations need to be noted for our study. Cemented fixation occurs within 10 minutes at room temperature, whereas cementless stems were press-fitted. The direct implications of our study can therefore only be observed in the postoperative phase, prior to bone in-growth in vivo; this, however, remains a clinically relevant period with registry data showing a PFF rate of 2.1% ≤ 90 days postoperatively (Lindberg-Larsen et al. 2017). Second, unidirectional compression force was used to produce a fracture, whereas fractures are always a result of a combination of forces. Our study also does not account for soft tissue contributions due to stripping of soft tissue and it therefore cannot precisely determine the mechanical effect of the implant in a patient where soft tissue is present. Finally, we used embalmed and not fresh frozen specimens. It has been shown, however, that the mechanical characteristics of fresh frozen specimens and embalmed cadaveric specimens are similar (Topp et al. 2012). A polyethylene-ceramic bearing has been used, as this is the standard bearing used in our institution. Even though the revision rate for this bearing is lower (Peters et al. 2018), we do not believe it affected the outcome of this particular study.

In summary, implanting a cemented version of the stem increases the load-to-failure force by 25%. We recommend using a cemented stem in older patients and patients who are at risk of falling.

## References

[CIT0001] Abdel M P, Watts C D, Houdek M T, Lewallen D G, Berry D J Epidemiology of periprosthetic fracture of the femur in 32 644 primary total hip arthroplasties: a 40-year experience. Bone Joint J 2016; 98-B(4): 461–7. 27037427 10.1302/0301-620X.98B4.37201

[CIT0002] Aro E, Alm J J, Moritz N, Mattila K, Aro H T Good stability of a cementless, anatomically designed femoral stem in aging women: a 9-year RSA study of 32 patients. Acta Orthop 2018; 89(5): 490–5. 29987941 10.1080/17453674.2018.1490985 PMC6202764

[CIT0003] Carli A V, Negus J J, Haddad F S Periprosthetic femoral fractures and trying to avoid them: what is the contribution of femoral component design to the increased risk of periprosthetic femoral fracture? Bone Joint J 2017; 99-B(1 Suppl. A): 50–9. 28042119 10.1302/0301-620X.99B1.BJJ-2016-0220.R1

[CIT0004] Carlson S W, Liu S S, Callaghan J J Not all cementless femoral stems are created equal but the results may be comparable. Bone Joint J 2017; 99-B(1 Suppl. A): 14–17. 28042113 10.1302/0301-620X.99B1.BJJ-2016-0269.R1

[CIT0005] Cook R E, Jenkins PJ, Walmsley P J, Patton J T, Robinson C M Risk factors for Periprosthetic Fractures of the Hip: A Survivorship Analysis. Clin Orthop Relat Res 2008; 466(7): 1652–6. 18470576 10.1007/s11999-008-0289-1 PMC2505237

[CIT0006] Erhardt J B, Khoo P P, Stoffel K K, Yates P J Periprosthetic fractures around polished collarless cemented stems: the effect of stem design on fracture pattern. Hip Int 2013; 23(5): 459–64. 23813164 10.5301/hipint.5000052

[CIT0007] Flatøy B, Röhrl S M, Bøe B, Nordsletten L No medium-term advantage of electrochemical deposition of hydroxyapatite in cementless femoral stems. 5-year RSA and DXA results from a randomized controlled trial. Acta Orthop 2016; 87(1): 42–7 26364953 10.3109/17453674.2015.1084768 PMC4940590

[CIT0008] Foster A P, Thompson N W, Wong J, Charlwood A P Periprosthetic femoral fracture: a comparison between cemented and uncemented hemiarthroplasties. Injury 2005; 36(3): 424–9. 15710161 10.1016/j.injury.2004.07.023

[CIT0009] Fottner A, Woiczinski M, Kistler M, Schröder C, Schmidutz T F, Jansson V, Schmidutz F Influence of undersized cementless hip stems on primary stability and strain distribution. Arch Orthop Trauma Surg 2017; 137(10): 1435–41. 28865042 10.1007/s00402-017-2784-x

[CIT0010] Ginsel B L, Morishima T, Wilson L J, Whitehouse S L, Crawford R W Can larger-bodied cemented femoral components reduce periprosthetic fractures? A biomechanical study. Arch Orthop Trauma Surg 2015; 135(4): 517–22. 25724411 10.1007/s00402-015-2172-3

[CIT0011] Hailer N P, Garellick G, Kärrholm J Uncemented and cemented primary total hip arthroplasty in the Swedish Hip Arthroplasty Register. Acta Orthop 2010; 81(1): 34–41. 20180715 10.3109/17453671003685400 PMC2856202

[CIT0012] Inngul C, Blomfeldt R, Ponzer S, Enocson A Cemented versus uncemented arthroplasty in patients with a displaced fracture of the femoral neck: a randomised controlled trial. Bone Joint J 2015; 97-B(11): 1475–80. 26530648 10.1302/0301-620X.97B11.36248

[CIT0013] Jämsen E, Eskelinen A, Peltola M, Mäkelä K High early failure rate after cementless hip replacement in the octogenarian. Clin Orthop Relat Res 2014; 472(9): 2779–89. 24771260 10.1007/s11999-014-3641-7 PMC4117887

[CIT0014] Kazi H A, Whitehouse S L, Howell J R, Timperley A J Not all cemented hips are the same: a register-based (NJR) comparison of taper-slip and composite beam femoral stems. Acta Orthop 2019; 90(3): 214–9. 30838914 10.1080/17453674.2019.1582680 PMC6534220

[CIT0015] Khanuja H S, Vakil J J, Goddard M S, Mont M A Cementless femoral fixation in total hip arthroplasty. J Bone Joint Surg Am 2011; 93(5): 500–9. 21368083 10.2106/JBJS.J.00774

[CIT0016] Kiran M, Johnston L R, Sripada S, Mcleod G G, Jariwala A C Cemented total hip replacement in patients under 55 years. Acta Orthop 2018;89(2): 152–5. 29345170 10.1080/17453674.2018.1427320 PMC5901511

[CIT0017] Klasan A, Sen A, Dworschak P, El-Zayat B F, Ruchholtz S, Schuettler K F, Schmitt J, Heyse T J Ten-year follow-up of a cemented tapered stem. Arch Orthop Trauma Surg 2018; 138(9): 1317–22. 30043147 10.1007/s00402-018-3002-1

[CIT0018] Langslet E, Frihagen F, Opland V, Madsen J E, Nordsletten L, Figved W Cemented versus uncemented hemiarthroplasty for displaced femoral neck fractures: 5-year followup of a randomized trial. Clin Orthop Relat Res 2014; 472(4): 1291–9. 24081667 10.1007/s11999-013-3308-9 PMC3940758

[CIT0019] Lehil M S, Bozic K J Trends in total hip arthroplasty implant utilization in the United States. J Arthroplasty 2014; 29(10): 1915–8. 25062807 10.1016/j.arth.2014.05.017

[CIT0020] Li M G, Rohrl S M, Wood D J, Nivbrant B Periprosthetic changes in bone mineral density in 5 stem designs 5 years after cemented total hip arthroplasty. No relation to stem migration. J Arthroplasty 2007; 22(5): 689–91. 17689776 10.1016/j.arth.2006.05.035

[CIT0021] Lindberg-Larsen M, Jørgensen C C, Solgaard S, Kjersgaard A G, Kehlet H, on behalf of the LFC for F-T, Group KRC Increased risk of intraoperative and early postoperative periprosthetic femoral fracture with uncemented stems. Acta Orthop 2017; 88(4): 390–4. 28290738 10.1080/17453674.2017.1302908 PMC5499329

[CIT0022] McMinn D J W, Snell K I E, Daniel J, Treacy R B C, Pynsent P B, Riley R D Mortality and implant revision rates of hip arthroplasty in patients with osteoarthritis: registry based cohort study. BMJ 2012; 344: e3319. 22700782 10.1136/bmj.e3319 PMC3375206

[CIT0023] Morishima T, Ginsel B L, Choy G G, Wilson L J, Whitehouse S L, Crawford R W Periprosthetic fracture torque for short versus standard cemented hip stems: an experimental in vitro study. J Arthroplasty 2014; 29(5): 1067–71. 24295802 10.1016/j.arth.2013.10.016

[CIT0024] Moskal J T, Capps S G, Scanelli J A Still no single gold standard for using cementless femoral stems routinely in total hip arthroplasty. Arthroplasty Today 2016; 2(4): 211–18. 28326430 10.1016/j.artd.2016.02.001 PMC5247516

[CIT0025] Palan J, Smith M C, Gregg P, Mellon S, Kulkarni A, Tucker K, Blom A W, Murray D W, Pandit H The influence of cemented femoral stem choice on the incidence of revision for periprosthetic fracture after primary total hip arthroplasty: an analysis of national joint registry data. Bone Joint J 2016; 98-B(10): 1347–54. 27694588 10.1302/0301-620X.98B10.36534

[CIT0026] Peters R M, Van Steenbergen L N, Stevens M, Rijk P C, Bulstra S K, Zijlstra W P The effect of bearing type on the outcome of total hip arthroplasty. Acta Orthop 2018; 89(2): 163–9. 29160130 10.1080/17453674.2017.1405669 PMC5901513

[CIT0027] Phillips J R A, Moran C G, Manktelow A R J Periprosthetic fractures around hip hemiarthroplasty performed for hip fracture. Injury 2013; 44(6): 757–62. 23103113 10.1016/j.injury.2012.09.015

[CIT0028] Sabharwal S, Wilson H, Reilly P, Gupte C M Heterogeneity of the definition of elderly age in current orthopaedic research. Springerplus. 2015; 4. 10.1186/s40064-015-1307-x PMC4573966 26405636

[CIT0029] Scheerlinck T, Casteleyn P-P The design features of cemented femoral hip implants. J Bone Joint Surg Br 2006; 88(11): 1409–18. 17075082 10.1302/0301-620X.88B11.17836

[CIT0030] Sheth N P, Brown N M, Moric M, Berger R A, Della Valle C J Operative treatment of early peri-prosthetic femur fractures following primary total hip arthroplasty. J Arthroplasty 2013; 28(2): 286–91. 22868075 10.1016/j.arth.2012.06.003

[CIT0031] Sidler-Maier C C, Waddell J P Incidence and predisposing factors of periprosthetic proximal femoral fractures: a literature review. Int Orthop 2015; 39(9): 1673–82. 25813458 10.1007/s00264-015-2721-y

[CIT0032] Thien T M, Chatziagorou G, Garellick G, Furnes O, Havelin L I, Mäkelä K, Overgaard S, Pedersen A, Eskelinen A, Pulkkinen P, Kärrholm J Periprosthetic femoral fracture within two years after total hip replacement: analysis of 437,629 operations in the Nordic Arthroplasty Register Association database. J Bone Joint Surg Am 2014; 96(19): e167. 25274795 10.2106/JBJS.M.00643

[CIT0033] Topp T, Müller T, Huss S, Kann P H, Weihe E, Ruchholtz S, Zettl R P Embalmed and fresh frozen human bones in orthopedic cadaveric studies: which bone is authentic and feasible? Acta Orthop 2012; 83(5): 543–7. 22978564 10.3109/17453674.2012.727079 PMC3488184

[CIT0034] Walpole SC, Prieto-Merino D, Edwards P, Cleland J, Stevens G, Roberts I The weight of nations: an estimation of adult human biomass. BMC Public Health 2012; 12: 439. 22709383 10.1186/1471-2458-12-439 PMC3408371

[CIT0035] Wright N C, Looker AC, Saag K G, Curtis J R, Delzell E S, Randall S, Dawson-Hughes B The recent prevalence of osteoporosis and low bone mass in the United States based on bone mineral density at the femoral neck or lumbar spine. J Bone Miner Res 2014; 29(11): 2520–6. 24771492 10.1002/jbmr.2269 PMC4757905

